# Case Report: Syphilitic Hepatitis–A Rare and Underrecognized Etiology of Liver Disease With Potential for Misdiagnosis

**DOI:** 10.3389/fmed.2021.789250

**Published:** 2021-11-29

**Authors:** Hiba A. Al Dallal, Siddharth Narayanan, Hanah F. Alley, Michael J. Eiswerth, Forest W. Arnold, Brock A. Martin, Alaleh E. Shandiz

**Affiliations:** ^1^Department of Pathology and Laboratory Medicine, University of Louisville, Louisville, KY, United States; ^2^Department of Pediatrics, Nationwide Children's Hospital, Columbus, OH, United States; ^3^Department of Neurology, University of Louisville, Louisville, KY, United States; ^4^Department of Internal Medicine, University of Louisville, Louisville, KY, United States; ^5^Division of Infectious Diseases, University of Louisville, Louisville, KY, United States

**Keywords:** syphilis, drug abuse, hepatitis, seizure, liver enzymes, infection

## Abstract

Syphilitic hepatitis (SH) in adults is a rare condition that can be easily misdiagnosed. Clinical and histopathologic manifestations of SH can mimic other infectious and non-infectious conditions, and the diagnosis should be considered in all at-risk patients with abnormal liver function tests. We present an unusual case of SH presenting with seizures and multiple liver lesions. This case report, in line with other newly published reports, promotes awareness of SH as a rare manifestation of treponemal infection and highlights the importance of including SH in the differential diagnosis for patients at risk for sexually transmitted infections and presenting with liver enzyme abnormalities. From a hospital quality control and socioeconomic perspective, our case adds to the growing body of evidence that demonstrates an increasing incidence of patients suffering from venereal diseases and injection drug use disorders, and the burden these conditions place on the healthcare system. Recognition of the clinicopathologic features of SH is required to prevent missed diagnosis and to foster systematic crosstalk between healthcare staff and public health personnel managing this problem.

## Introduction

Syphilis is a disease caused by the non-hepatotropic bacterium *Treponema pallidum* and is associated with high-risk sexual activity. The stages of syphilis are well-defined, but the clinical manifestations can greatly vary between these stages (1, 2). In comparison to the first two stages (primary/secondary), untreated tertiary syphilis can present years after initial infection to cause devastating multi-organ system manifestations (3). While the overall incidence of syphilitic hepatitis (SH) is low, SH may occur in an estimated 3% of secondary syphilis cases (1). The incidence of tertiary syphilis presenting as SH is much less common.

Syphilitic hepatitis is usually asymptomatic or presents with non-specific symptoms. Non-hepatic manifestations of secondary syphilis, such as a characteristic diffuse maculopapular rash, are usually more helpful in pointing to the diagnosis than any hepatic signs or symptoms. The diagnosis of SH typically requires biochemical evidence of liver injury in the setting of confirmed treponemal serology, after exclusion of alternative causes of hepatic dysfunction (4). The pattern of liver enzyme abnormalities is often cholestatic with altered alkaline phosphatase (ALP) levels and mildly elevated transaminases and bilirubin. Liver biopsy is often not required for diagnosis as response to antimicrobial therapy serves as a useful confirmatory finding.

We present an unusual case of an incarcerated adult female with a history of injection drug use (IDU) and multiple sexual partners who presented to our hospital with a constellation of neurological deficits and multiple liver lesions on imaging. After a comprehensive workup, she was diagnosed with tertiary SH with hepatic gummatous lesions but left against medical advice after only completing 4 days of penicillin treatment.

## Case Report

**Figure 1:**
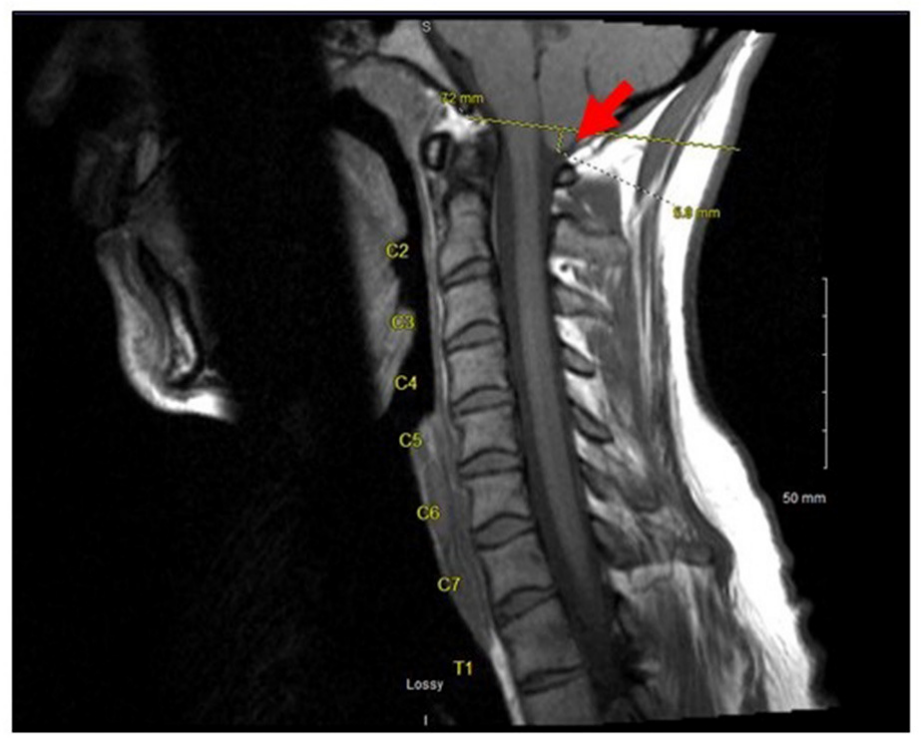
A magnetic resonance imaging of the cervical spine showing a Chiari I malformation (red arrow) with a 5.8 mm displacement of the cerebellar vermis through the foramen magnum.

A 36-year-old incarcerated female with a history of polysubstance abuse and multiple sexual partners was found exhibiting seizure-like activity in her cell. On arrival at our hospital, she was unresponsive with convulsions of all extremities. Upon physical examination, she had upper motor neuron symptoms, including dilated pupils, rigidity of the lower extremities, and clonus, and a rectal temperature of 103°F. The patient was administered benzodiazepine to control the convulsions and intubated for airway protection. A computerized tomography (CT) scan of her head and neck was unremarkable, and magnetic resonance imaging (MRI) of the cervical spine demonstrated Chiari I malformation (Figure 1). An electroencephalogram of the brain confirmed seizure activity. Chest radiographs showed no evidence of an acute pulmonary process. Based on the initial presentation, a broad differential diagnosis was considered, including drug/toxin-induced neurological manifestations, infection, paraneoplastic syndrome, and metabolic (e.g., vitamin B12) deficiency.

An extensive workup was performed that included complete blood count, liver function tests, toxicology screens, and testing for multiple infectious diseases. Her white blood cell count was highly elevated (16 × 10^9^ cells/L). Liver function tests revealed mildly elevated aspartate aminotransaminase (35 units/L, normal 8–34 units/L), alanine aminotransaminase (47 units/L, normal 7–24 units/L), and alkaline phosphatase (112 units/L, normal 25–105 units/L) with normal bilirubin. Vitamin B12 level was within the normal range. Urine toxicology was positive for cannabinoids, amphetamines, and benzodiazepines. An HIV screen was negative; however, the patient tested positive for *Treponema pallidum* antibodies (titer of 1:1,024) as well as hepatitis B surface antigen and DNA (PCR confirmation). A lumbar puncture for a cerebrospinal fluid culture and analysis was deferred due to the risk of herniation because of her Chiari I malformation.

**Figure 2:**
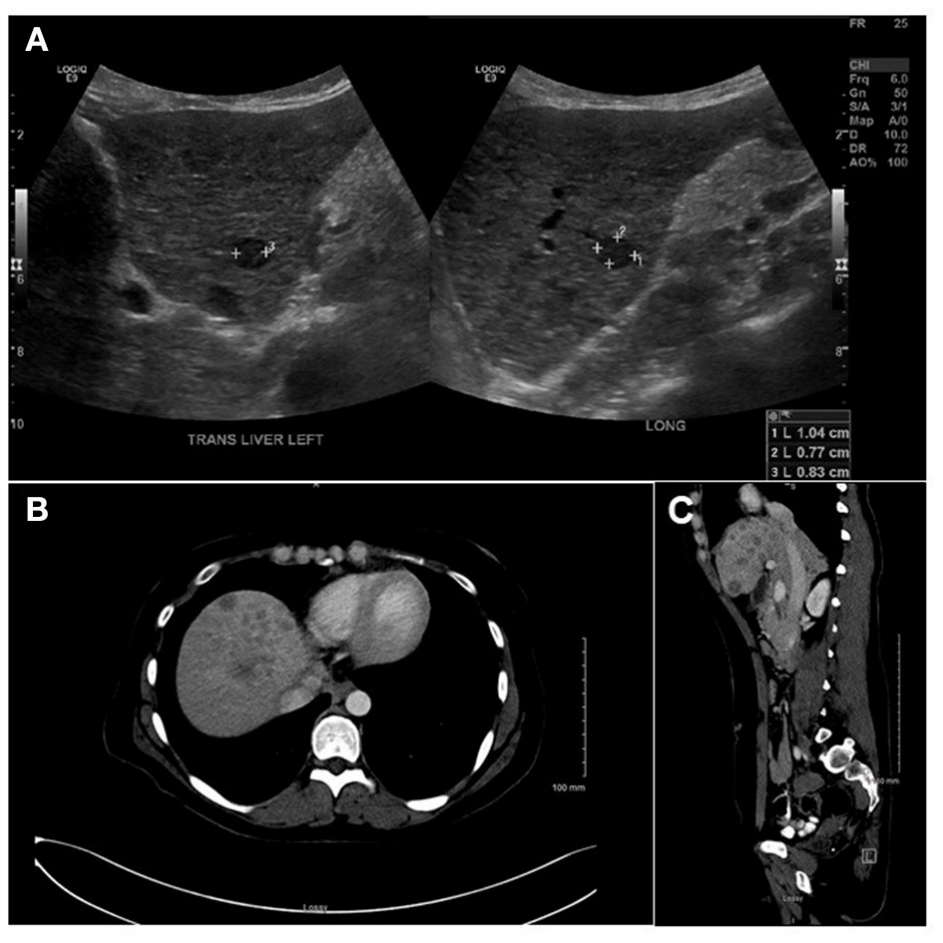
**(A)** Liver ultrasound showing multiple hypo-echoic lesions ranging between 0.5 and 3.3 cm. **(B)** A liver computerized tomography scan showing multiple hypo-echoic lesions with peripheral rim of increased enhancement. **(C)** A magnetic resonance imaging (sagittal view) showing same as **(A,B)**.

Empiric treatment with intravenous penicillin G was initiated for a presumed diagnosis of neurosyphilis. However, the patient continued to exhibit unexplained, medically refractory severe nausea and vomiting, prompting abdominal imaging. An ultrasound assessment revealed multiple bilobar hypoechoic hepatic lesions (Figure 2A) which were confirmed as multiple hypoenhancing lesions on subsequent abdominal CT and MRI (Figures 2B,C). These additional imaging results raised consideration for atypical hepatic abscesses vs. metastatic malignancy, and a targeted liver core biopsy was performed.

**Figure 3:**
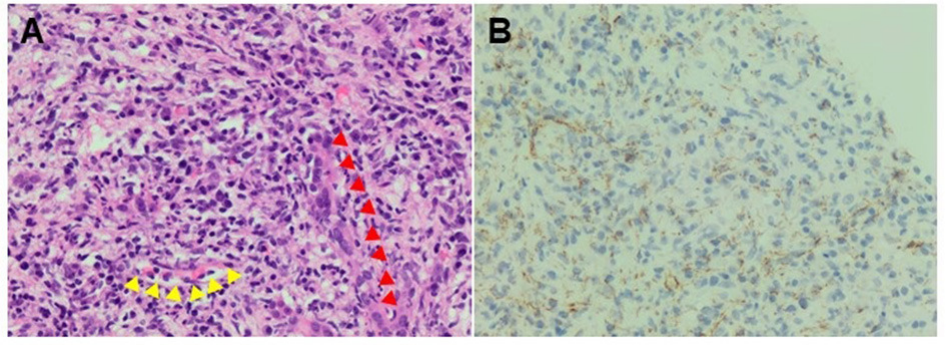
**(A)** A H&E stain of the liver biopsy (400 X) showing mixed inflammatory infiltrate composed predominantly of lymphocytes and plasma cells and smaller numbers of neutrophils and eosinophils. The bile duct (red arrows) and portal vessel (yellow arrows) are highlighted. **(B)** An Anti-*Treponema pallidum* immunohistochemical staining confirmed the presence of spirochetes and highlighted numerous microorganisms having an epitheliotropic and vasculotropic pattern. The IHC was performed on the same tissue area as for the H&E stain.

Histologic sections of the biopsy showed involvement of the liver parenchyma by a dense mixed lymphoplasmacytic and granulocytic inflammatory infiltrate associated with reactive fibrosis (Figure 3A), foci of necrosis, and clusters of epithelioid histiocytes, consistent with inflammatory pseudo-tumor. In addition, foci of vascular and biliary inflammatory injury were present (Figure 3A). While a Warthin-Starry stain on the sample was negative, immunohistochemistry for treponemal organisms confirmed the presence of *Treponema pallidum* (Biocare APA 135 AA) and the diagnosis of tertiary stage SH with hepatic gummas (Figure 3B). The patient's liver function tests were monitored daily and showed initial improvement (Table 1); however, she left against medical advice following completion of only 4 days of the recommended 10–14-day course of intravenous penicillin for neurosyphilis.

**Table 1:** Liver biochemical profile of our patient before and after antimicrobial treatment.

**Liver function test**	**Hospital: Day 0**	**Hospital: Day 13 (discharge)**
ALP (normal: 25–105 units/L)	112	84
AST (normal: 8–34 units/L)	35	29
ALT (normal: 7–24 units/L)	47	47
Albumin (normal: 3.4–4.8 g/dL)	2.7	3.1
Total bilirubin (normal: 0.2–1.1 mg/dL)	0.6	0.5

*ALP, alkaline phosphatase; AST, aspartate transaminase; ALT, alanine transaminase*.

## Discussion

Syphilis constantly challenges healthcare providers due to its multi-organ involvement, overlapping clinical stages, protean presentation, and it is an under-recognized etiology of liver dysfunction. In a retrospective study, liver enzyme abnormalities were common in patients with early syphilis (39%), yet only a small fraction (2.7%) were diagnosed with SH (5). Some authors have previously proposed clinical diagnostic criteria for SH (6), although diagnosis is still limited by the known clinical heterogeneity and lack of specific features of the disease (7).

The clinical manifestations of SH tend to be non-specific in adults. Common symptoms including low-grade fever, abdominal pain, sore throat, headache, weight loss, arthralgia or myodynia, splenomegaly, lymphadenopathy, and uveitis (7). Moderate elevations in liver enzymes, as well as markedly increased ALP and gamma-glutamyl transpeptidase levels, are reported in patients with SH (1, 2, 4). While the classic symmetric maculopapular rash of secondary syphilis, which involves the trunk and extremities including palms and soles, is a strong clue to the diagnosis of SH as a cause of liver enzyme abnormalities (8), it is not uniformly observed in all patients. Liver involvement represents spirochete dissemination (9) but can occur at any stage of the disease. In a systematic review of SH, the vast majority of patients (88.9%) presented with early stage (i.e., primary or secondary) disease; whereas, a small minority presented with latent (4.9%) or tertiary (6.3%) stage disease (10) when classic signs and symptoms of syphilis may be less apparent.

The histopathologic features of SH are likewise non-specific. Cases typically show mixed lymphoplasmacytic and granulocytic portal inflammation with variable bile duct injury and associated granulomas (9). A vasculotropic and epitheliotropic pattern of inflammation may be seen in some cases, but this is not etiologically specific and overlaps with other, more common inflammatory liver diseases. Although identification of spirochetes in the tissue by special stain or immunohistochemistry is diagnostic in the appropriate setting (11), false negative staining is not uncommon in the setting of disseminated treponemal disease, reaffirming the need for close clinicopathologic correlation to make the diagnosis in most cases. In the absence of known or reported risk factors for SH, the diagnosis is likely to be missed. Furthermore, potentially misleading clinical and imaging findings may prompt consideration and additional testing for other infectious, autoimmune, or neoplastic diseases, leading to delayed or misdiagnosis.

Our case emphasizes the importance of maintaining a broad differential diagnosis for hepatic enzyme abnormalities, including a comprehensive review of patient risk factors for common and uncommon causes of hepatitis. Despite an initial presentation with only mild liver enzyme elevations and multiple confounding and potentially misleading clinical and imaging findings, careful clinicopathologic correlation and appropriate diagnostic investigation eventually facilitated a conclusive diagnosis of tertiary SH.

Most cases of SH resolve with antibiotic medications for treponemal infection. Heightened clinical awareness and recognition of patients at risk for the disease are necessary to facilitate timely diagnosis and treatment. Risk factors including unprotected sexual activity, polysubstance abuse and IDU attribute to notably high rates of coinfection, increased hospital costs and a massive burden on the healthcare system (2, 12, 13). The incidence of primary and secondary syphilis are on the rise (1), and the identification and treatment of syphilis cases prevents progression to tertiary syphilis in affected individuals and the spread to other individuals. Therefore, increased attention to the diagnosis of SH by care providers and pathologists is essential for achieving positive patient outcomes as well as hospital quality control and socioeconomic goals.

## Conclusions

SH can be easily overlooked and misdiagnosed because of non-specific signs and symptoms at the presentation and mild hepatic manifestations, in addition to non-specific and overlapping histopathologic findings. The diagnosis of tertiary SH is particularly problematic as other clinical signs and symptoms of disseminated treponemal disease may be absent or inapparent. Given the availability of effective treatment, timely diagnosis can greatly decrease morbidity and mortality associated with this condition. Even in the absence of non-hepatic manifestations, SH should be considered in high-risk patients with altered liver function tests. Multi-disciplinary efforts from healthcare and regulatory bodies are needed to reduce its growing incidence.

## Data Availability Statement

The raw data supporting the conclusions of this article will be made available by the authors, without undue reservation.

## Ethics Statement

This case report was reviewed by the IRB and determined that it did not meet the common rule requirements and was deemed Non-Human Subjects Research (NHSR). No personal health identifiers are included in this case report. The patient consent was obtained.

## Author Contributions

HAA collected the data and interpreted the diagnosis. SN analyzed the literature and wrote the manuscript. HFA and ME helped in collecting the data and edited the manuscript. FA, BM, and AS provided framework for the study, reviewed, and edited the manuscript. All authors have read and agreed to the final version of the manuscript.

## Author Disclaimer

The findings and conclusions in this manuscript are those of the authors alone and do not necessarily represent the official position of the Centers for Disease Control and Prevention.

## Conflict of Interest

The authors declare that the research was conducted in the absence of any commercial or financial relationships that could be construed as a potential conflict of interest.

## Publisher's Note

All claims expressed in this article are solely those of the authors and do not necessarily represent those of their affiliated organizations, or those of the publisher, the editors and the reviewers. Any product that may be evaluated in this article, or claim that may be made by its manufacturer, is not guaranteed or endorsed by the publisher.

## References

[1] Alemam A Ata S Shaikh D Leuzzi B Makker J. Syphilitic hepatitis: a rare cause of acute liver injury. Cureus. (2021) 13:e14800. 10.7759/cureus.14800 34094758 PMC8168444

[2] Narang N Al-Jashaami L Patel N. Spirochetes in the liver: an unusual presentation of a common STI. Case Rep Med. (2019) 2019:1012405. 10.1155/2019/1012405 31885599 PMC6927064

[3] Ghanem KG Ram S Rice PA. The modern epidemic of syphilis. N Engl J Med. (2020) 382:845–54. 10.1056/NEJMra1901593 32101666

[4] Horn CL Jalali S Abbott J Stein M. A surprising diagnosis: syphilitic gastritis and hepatitis. Am J Med. (2018) 131:1178–81. 10.1016/j.amjmed.2018.03.023 29653086

[5] Adachi E Koibuchi T Okame M Sato H Kikuchi T Koga M. Liver dysfunction in patients with early syphilis: a retrospective study. J Infect Chemother. (2013) 19:180–2. 10.1007/s10156-012-0440-5 22692597

[6] Mullick CJ Liappis AP Benator DA Roberts AD Parenti DM Simon GL. Syphilitic Hepatitis in HIV-Infected Patients: A Report of 7 Cases and Review of the Literature. Clin Infect Dis. (2004) 39:e100–5. 10.1086/425501 15546070

[7] Huang J Lin S Wang M Wan B Zhu Y. Syphilitic hepatitis: a case report and review of the literature. BMC Gastroenterol. (2019) 19:191. 10.1186/s12876-019-1112-z 31744461 PMC6862847

[8] Kaya A Kaya SY. Management of syphilitic hepatitis. BMC Gastroenterol. (2020) 20:379. 10.1186/s12876-020-01496-5 33183229 PMC7664093

[9] Pizzarossa AC Rebella M. Hepatitis in patients with syphilis: an overlooked association. BMJ Case Rep. (2019) 12:226918. 10.1136/bcr-2018-226918 30696640 PMC6350734

[10] Huang J Lin S Wan B Zhu Y. A systematic literature review of syphilitic hepatitis in adults. J Clin Transl Hepatol. (2018) 6:306–9. 10.14218/JCTH.2018.00003 30271743 PMC6160304

[11] Hoang MP High WA Molberg KH. Secondary syphilis: a histologic and immunohistochemical evaluation. J Cutan Pathol. (2004) 31:595−9. 10.1111/j.0303-6987.2004.00236.x 15330990

[12] Elnazeir M Narayanan S Badugu P Hussain A Stephens CB Bhagat R. Neurological manifestations associated with synthetic cannabinoid use–a case series. Open Neurol J. (2020) 14:53–8. 10.2174/1874205X02014010053

[13] Al Dallal HA Narayanan S Jones CM Lockhart SR Snyder JW. First case report of an unusual fungus (sporopachydermia lactativora) associated with a pulmonary infection in a drug injection user. Clin Pathol. (2021) 14:2632010X211029970. 10.1177/2632010X211029970 34345817 PMC8280816

